# Comments on “Ileosigmoid knot in a patient with Down syndrome: a unique surgical emergency” by Boussaidane S *et al*. (Pan Afr Med J. 2021;38: 8)

**DOI:** 10.11604/pamj.2021.38.360.28834

**Published:** 2021-04-14

**Authors:** Sabri Selcuk Atamanalp

**Affiliations:** 1 Department of General Surgery, Faculty of Medicine, Ataturk University, Erzurum, Turkey

**Keywords:** Ileosigmoid knotting, mental retardation, surgery

## Abstract

Ileosigmoid knotting (ISK) is the wrapping of ileum or sigmoid colon around the other structure causing a double-loop intestinal obstruction. Both ISK and ISK complicating mental retardation are very rare clinical entities. In this commentary, I would like to discuss some details of ISK complicating mental retardation regarding to a published paper in The Pan African Medical Journal.

## Commentary

I read with great interest the article titled “Ileosigmoid knot in a patient with Down syndrome: a unique surgical emergency” written by Boussaidane *et al*. [1]. Although ISK is a very rare clinical entity with a few hundred cases reported to date [2], it is relatively common in eastern Turkey, our practicing area. We have an 80-case experience with ISK over a 54.5-year period (between June 1966 and January 2021), which is one of the largest single-center ISK series over the world [2]. In the light of this experience, I would like to discuss the pathophysiology, diagnosis, classification, and treatment of ISK complicating mental retardation regarding to the case presented by the authors.

First, ISK in mental retarded patients is a very rare clinical entity [2]. For this reason, the case presented by the authors, ISK complicating Down syndrome, is extremely interesting. In our series, there was only one retarded patient (1.3%). Although mental retardation is thought as a predisposing factor in the development of ISK, most likely due to its rarity, the relationship between the mental retardation and ISK is not well explored [2]. In mental retarded patients, abnormal dietary habits such as overeating and irregular defecation practices such as chronic constipation may cause the elongation of sigmoid colon [3], which is known as an important anatomical prerequisite for ISK [2]. Similarly, psychotropic drugs, in addition to laxatives and enemas, which are used in patients with mental retardation, may lead to the discussed anatomopathology [3].

Second, although abdominal pain, distention, and obstipation, which are known as classical volvulus triad, suspect ISK and abdominal X-ray radiography helps to diagnosis by demonstrating coffee been sign, they generally cause an underdiagnosis by directing to nonspecific intestinal obstruction [4,5], as was presented in the authors´ case. Instead, computed tomography (CT), which demonstrates mesenteric whirl sign, a pathognomonic data of volvulus, is more diagnostic [4], as was indicated in the presented case. According to my experience, the preoperative diagnostic accuracy increased from 15-20% to 90% in ISK due to the usage of CT by 2000s.

Third, in past, many different classifications have been documented for ISK evaluating prevalence (sporadic or endemic), clinical course (acute, subacute, chronic), active component (ileum or sigmoid colon), volvulus direction (clockwise or counterclockwise), and volvulus degree (<360, 360, or >360) [6,7]. Among these, active component is the most frequently used rating and according to this classification, ileum active form (Type I) is the most common type as was seen in the authors´ case. Nevertheless, in my experience, to determine the active component may be difficult or impossible in some cases. Additionally, neither active component classification nor the others give sufficient information about the management or prognosis of ISK. For this reason, I prefer to use a new classification system, which directs the treatment and estimates the prognosis by evaluating age, American Society of Anesthesiologists (ASA) score, and local bowel conditions [8].

Last, following the resection of gangrenous bowel segment, a stoma generally saves the life [9,10]. Nevertheless, if a stoma is needed in ISK with both ileum and sigmoid colon gangrene, contrary to the authors´ choice including two-segment stoma, I prefer to perform only one stoma. It is clear that, when a complete ileostomy is performed, intestinal content doesn´t pass to colonic segments and large bowels discharge only mucosal secretion. In such cases, to perform a stoma in a single segment, in which poor bowel conditions (significant edema, borderline ischemia or difference in proximal and distal bowel ends) are present, may be the optimal choice and if possible, I prefer and advise to perform a colostomy instead of an ileostomy [8].

I congratulate the authors for their interesting presentation and look forward to their reply on my comments.

## References

[1] Boussaidane S, Samlali A, Hamri A, Narjis Y, Benelkhaiat BR (2021). Ileosigmoid knot in a patient with Down syndrome: a unique surgical emergency. Pan Afr Med J.

[2] Atamanalp SS (2018). Ileosigmoid knotting: One of the largest single-center series. Pak J Med Sci.

[3] Avots-Avotins KV, Waugh DE (1982). Colon volvulus and geriatric patient. Surg Clin N Am.

[4] Nazir S, Munir A, Sulman S, Waleed QM, Muhammad AY (2020). Ileosigmoid knot: A case report. J Liaquat Uni Med Health Sci.

[5] Mbanje C, Mungazi SG, Muchuweti D, Mazingi D, Mlothswa M, Maunganidze AJV (2020). Ileo-sigmoid knotting: the Parirenyatwa hospital experience. South Afr J Surg.

[6] Alver O, Oren D, Tireli M, Kayabasi B, Akdemir D (1993). Ileosigmoid knotting in Turkey. Dis Colon Rectum.

[7] Gupta AK, Ansari AM, Jayant S, Goel S, Bansal LK (2020). Ileosigmoid knotting causing double-lumen acute intestinal obstruction and gangrene-Review and a case report. J Clin Diagn Res.

[8] Atamanalp SS (2021). Ileosigmoid knotting: An update for Atamanalp classification. Pak J Med Sci.

[9] Abebe K, Sherefa K, Teshome H, Abebe E (2020). Ileosigmoid knotting: Analysis of patients clinical profiles and determinants of outcomes. Surg Res Pract.

[10] Atamanalp SS (2014). Treatment for ileosigmoid knotting: a single-center experience of 74 patients. Tech Coloproctol.

